# Multi-factor mediated functional modules identify novel classification of ulcerative colitis and functional gene panel

**DOI:** 10.1038/s41598-021-85000-3

**Published:** 2021-03-11

**Authors:** Lijie Lai, Hanyang Li, Qi Feng, Jun Shen, Zhihua Ran

**Affiliations:** 1 Division of Gastroenterology and Hepatology, Key Laboratory of Gastroenterology and Hepatology, Ministry of Health, Inflammatory Bowel Disease Research Center, Renji Hospital, School of Medicine, Shanghai Jiao Tong University, Shanghai Institute of Digestive Disease, 160# Pu Jian Ave, Shanghai, 200127 China; 2 Department of Radiology, Renji Hospital, School of Medicine, Shanghai Jiao Tong University, 160 Pu Jian Road, Shanghai, 200127 China

**Keywords:** Classification and taxonomy, Ulcerative colitis

## Abstract

Ulcerative colitis is a chronic, idiopathic, and inflammatory disease of the rectal and colonic mucosa, the behavior of which is of heterogeneity in individuals. Here, we explored the multifactor-mediated functional modules associated with ulcerative colitis classification in the whole genome. Datasets downloaded from the GEO database were used to identify differentially expressed genes between ulcerative colitis patients and healthy individuals initially, followed by acquisition of the remaining ulcerative colitis -related genes from the OMIM and STRING databases. The results identified 914 ulcerative colitis-related genes, of which 60 were differentially expressed genes obtained from GEO datasets. Through weighted co-expression network analysis of ulcerative colitis-related genes, four modules were obtained, three of which were related to ulcerative colitis. Following interactions between microRNA, long noncoding RNA, transcription factors, and module hub genes were predicted and used to construct ulcerative colitis multifactor networks. Additionally, we performed consensus clustering of the ulcerative colitis samples. The results revealed that ulcerative colitis could be divided into four subtypes, with six hub genes identified as potential biomarkers for classification. These findings offer novel insights into ulcerative colitis and a basis for disease classification of ulcerative colitis.

## Introduction

Ulcerative colitis (UC), a subtype of inflammatory bowel disease (IBD), is a chronic, relapsing, and nonspecific inflammatory disease, the etiology and pathogenesis of which are not fully understood. UC lesions are confined to the mucosa and submucosa and mostly located in the sigmoid colon and rectum but can also extend to the descending colon or even the entire colon. Typical clinical manifestations include diarrhea, purulent stool, and abdominal pain^1^. In Asia, UC incidence is much lower than that in Europe, with at incidence of 6.3 per 100,000 person-years^2^; however, the incidence has increased annually^3^.

Due to the heterogeneous and varying disease course, UC classification is critical for clinical management of patients. The most commonly used subclassification system for UC incorporates an assessment of disease extent and the severity of an individual relapse of the disease. Additionally, the Montreal classification of disease extent of UC can be divided into three subtypes: ulcerative proctitis, left-sided UC, and extensive UC^4^. The development of high-throughput microarray has allowed gene-expression profiling to identify genes associated with the clinical phenotype of UC. Alterations in gene expression in IBD patients correlate with the clinical phenotype^5^. Notably, several genes have been identified as biomarkers of the UC phenotype, including *polymeric immunoglobulin receptor*, *interleukin* (*IL*)-*8*, and *HLA class II histocompatibility antigen DRB1 beta chain*^6,7^. Although identification of differentially expressed genes (DEGs) is necessary, determining their interconnectedness is also important. Correlation networks are increasingly being used in bioinformatics applications, with weighted co-expression network analysis (WGCNA) commonly used to describe molecular mechanisms and reconstruct co-expression networks of genes in different samples^8^. MicroRNAs (miRNAs), transcription factors (TFs) and long noncoding RNAs (lncRNAs) also play roles in disease behavior of UC. Three mechanisms have been described for miRNA-specific gene regulation: translation repression, direct mRNA degradation, and miRNA-mediated mRNA decay^9^. Additionally, TFs and lncRNAs are capable of regulating gene expression, with lncRNAs also capable of interacting with miRNA. Therefore, it is necessary to study the multifactor-mediated gene modules to allow UC classification.

The Montreal classification offers a clinical view of ulcerative colitis classification. To obtain additional genetic information for supplementary clinical disease characteristics, we obtained UC-related modules via WGCNA, constructed a multifactor network of the functional modules, and explored potential functional modules and gene biomarkers useful for UC classification.

## Materials and methods

### Data resources

Two UC gene-expression datasets (GSE109142 and GSE111889)^10,11^ and a miRNA dataset (GSE133059) specific to UC were downloaded from the GEO database (https://www.ncbi.nlm.nih.gov/geo/) (Table 1). All datasets were open-accessed and no human data or animal experiments were included in the study. Therefore, there is no ethical approval problem in this study.

**Table 1:** UC datasets sample information.

Sample	Features	Control	Patient	All
**mRNA**				
GSE109142	Rectum	20	206	226
GSE111889	Rectum	23	27	50
	Ileum	21	24	45
**miRNA**				
GSE133059	Mucosa	8	8	16

### DEG analysis

Data from GSE109142 was log_2_ transformed, and differential expression analysis was performed using the limma package (http://www.bioconductor.org/packages/release/bioc/html/limma.html). R Software used in this study is R version 3.6.3 (Holding the Windsock) released on 2020-02-29. Genes showing significant differential expression (*p* < 0.05) and a log fold change (|log_2_FC|) > 2 were designated as significant genes. Analysis of GSE111889 was performed using DESeq2^12^, DEGs were identified according to the same p-value cut-off and a |log_2_FC|> 1. The miRNA dataset GSE133059 was preprocessed and normalized using the robust multi-array average method, followed by the Limma package for differential expression analysis. Differentially expressed miRNAs were selected according to a *p* < 0.05 and a |log_2_FC|> 2.

### UC-related genes

The STRING database (http://string-db.org/) was used to analyze gene interactions^13^. Submission of the 60 identified DEGs to STRING returned 744 interaction genes according to a minimum required interaction score of 0.9. We then downloaded 110 genes related to UC from the OMIM database (https://omim.org/)^14^. A total of 914 potential UC-related genes were obtained.

### WGCNA

UC-related modules were analyzed using WGCNA, which is used to describe the gene-association pattern between different samples. WGCNA can be used to identify gene sets with highly synergistic variation and identify candidate biomarkers or therapeutic targets based on the interconnectivity of gene sets and the association between gene sets and phenotypes. WGCNA of UC-related genes was performed using the R package WGCNA (https://cran.rstudio.com/web/packages/WGCNA/).

### GO and KEGG pathway enrichment analyses

Functional enrichment analysis was performed using ClueGO and CluepeDia^14^. A *p* < 0.05 and a kappa score ≥ 0.95 were set as cut-offs for analysis of each module.

### Identification of hub module genes

To identify the hub genes related to UC traits, we input the genes of each module into STRING (https://string-db.org/) and obtained a protein–protein interaction (PPI) network with default parameter values. We used the cytoHubba plugin (http://apps.cytoscape.org/apps/cytohubba)^15^, which integrates 11 topological analysis methods and six centralities with the Maximal Clique Centrality (MCC) algorithm, to explore the top 10 candidates as hub genes in the PPI network. The STRING database was further utilized for gene-interaction analysis of the hub genes of the four modules, with the network subsequently visualized using Cytoscape software (https://cytoscape.org/)^16^.

### Interaction between miRNA, lncRNA, TF and mRNA

Interactions between miRNA and mRNA were determined using miRwalk (http://mirwalk.umm.uni-heidelberg.de/)^17^, with these screened and confirmed as relevant according to at least three databases [miRwalk, TargetScan (http://www.targetscan.org/vert_72/), miRDB (http://mirdb.org/), and miRTarBase (http://mirtarbase.mbc.nctu.edu.tw/php/index.php)]^18,19^. MiRNA–lncRNA and TF–mRNA interactions were determined using StarBase (v.3.0; http://starbase.sysu.edu.cn) and TRRUST (v.2.0; http://www.grnpedia.org/trrust)^20,21^, respectively.

### Mapping hub genes to samples and UC classification analysis

Disease classification of UC was performed using hub genes. Consensus clustering of UC samples was performed using the R package ConsensusClusterPlus (https://bioconductor.org/packages/release/bioc/html/ConsensusClusterPlus.html). One-way analysis of variance (ANOVA) was then performed to obtain the hub genes with significantly different expression levels in each subtype (*p* < 0.05).

## Results

### Identification of DEGs between UC samples and normal samples

**Figure 1:**
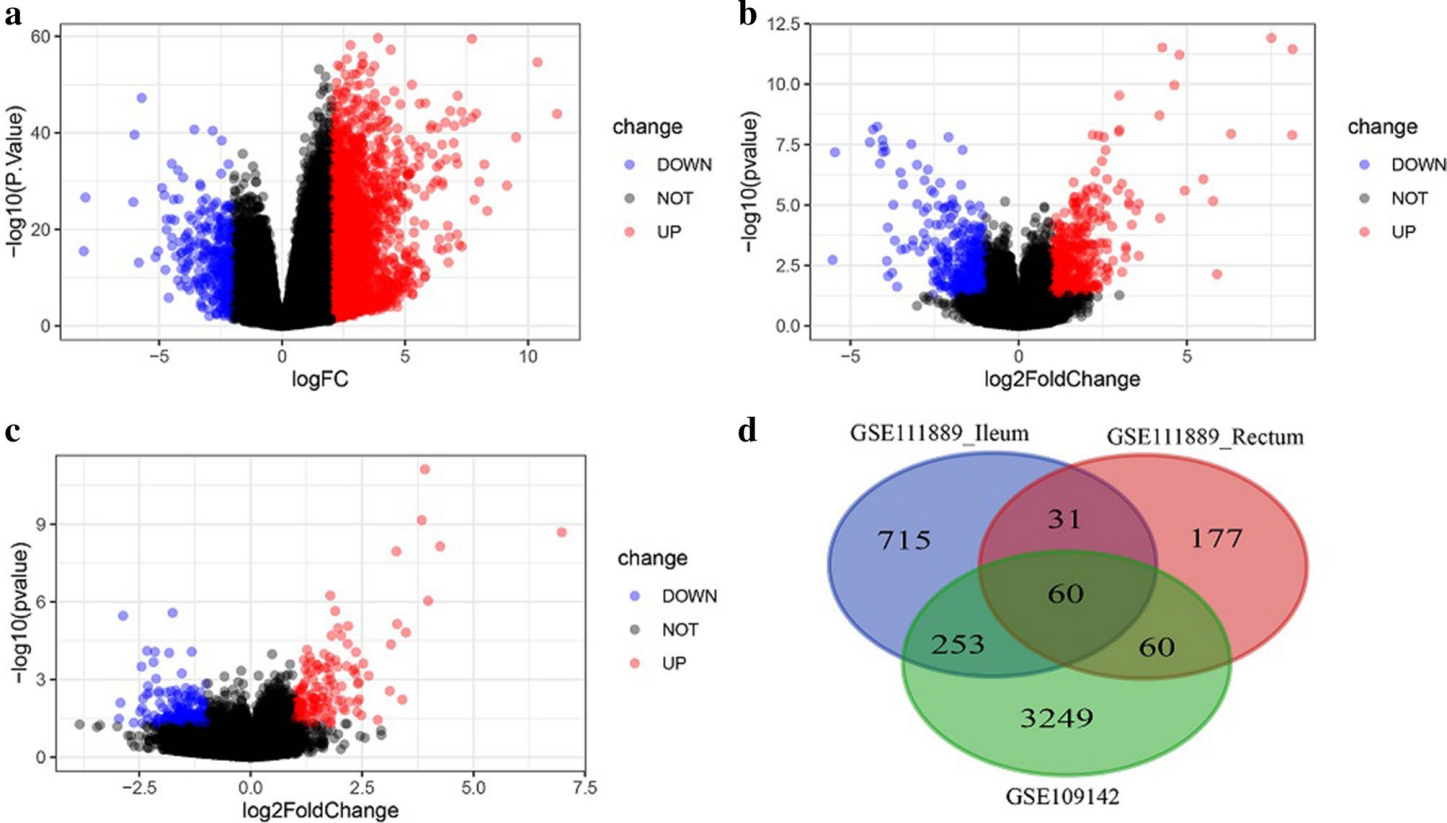
The differential expression analysis results in Volcano Plot and Venn diagram. (**a**) shows the differential expression analysis results of GSE109142 dataset. (**b**,**c**) show the differential expression analysis results of GSE111889 dataset. (**d**) shows Venn diagram of the DEGs in three groups.

The GSE109142 dataset contains data for 226 rectal samples data, of which 20 are from healthy controls and 206 from UC patients. A total of 3,622 DEGs were obtained by differential expression analysis, among which 3,287 were upregulated and 335 downregulated (Fig. 1a, Supplementary Table 1). The GSE111889 dataset includes data from 45 ileum samples (21 healthy controls and 24 UC patients) and 50 rectal samples (23 health controls and 27 UC patients). Differential expression analysis of the ileum samples identified 1,059 DEGs, of which 611 were upregulated and 448 downregulated (Fig. 1b, Supplementary Table 2). Analysis of rectal specimens identified 328 DEGs, of which 148 were upregulated and 180 downregulated (Fig. 1c, Supplementary Table 3). Plotting the identified DEGs revealed 60 commons among the three groups (Fig. 1d).

### WGCNA

**Figure 2:**
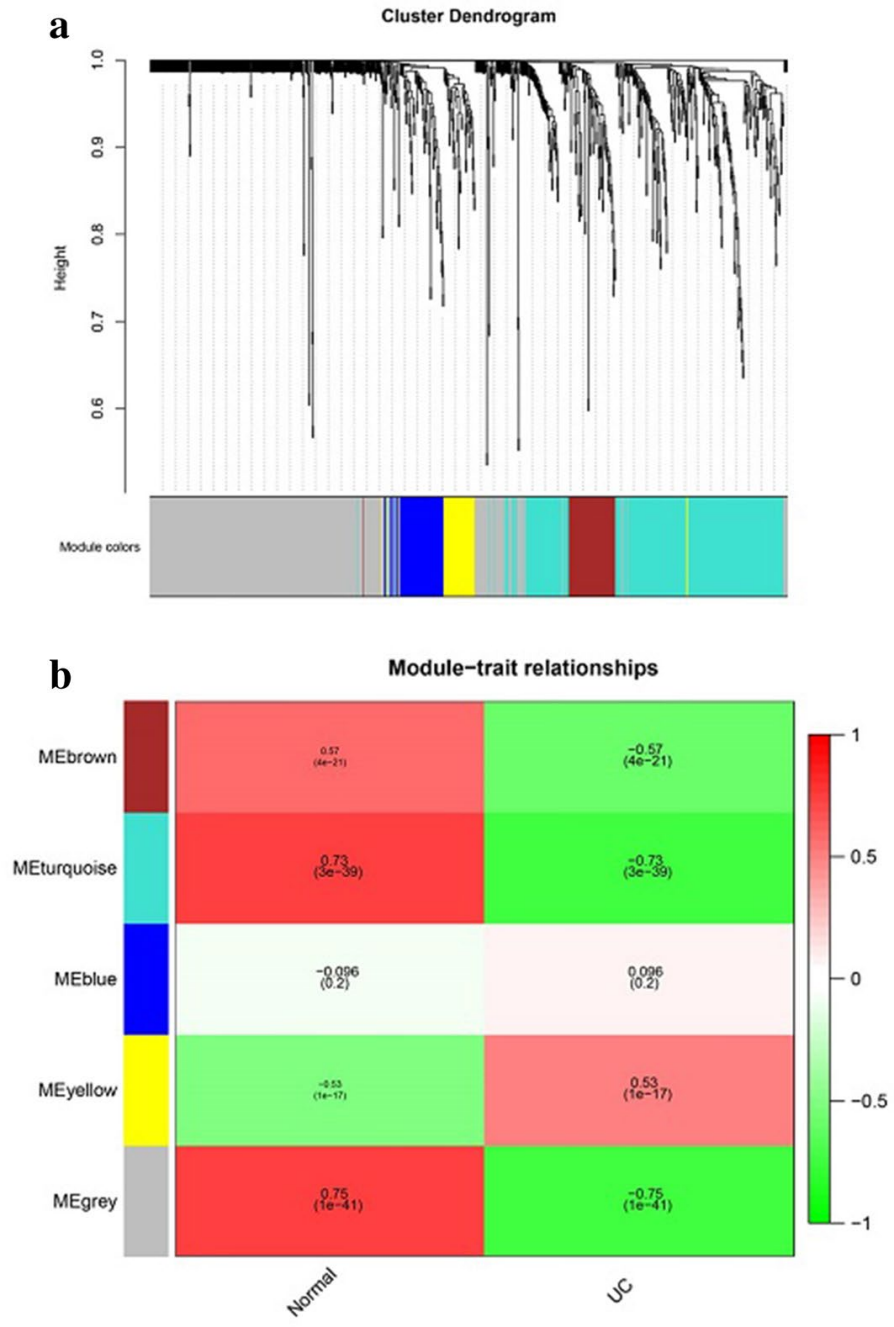
(**a**) shows the cluster dendrogram of UC-related modules, including five modules. (**b**) shows the heatmap of module-trait relationships made by WGCNA R package (https://cran.rstudio.com/web/packages/WGCNA/).

The 914 UC-related genes were mapped to the gene-expression profile of GSE109142, and scale-free WGCNA was performed. We identified five modules (grey, turquoise, blue, brown, and yellow) using default parameters (Fig. 2a, Supplementary Table 4). Among these, genes in the grey module represented those not been assigned to other modules. Genes in the brown and turquoise modules were significantly positively correlated with healthy individuals and negatively correlated with UC patients, whereas those in the yellow modules were significantly negatively correlated with healthy individuals and positively correlated with UC patients (Fig. 2b).

### GO and KEGG analyses of module genes

**Figure 3:**
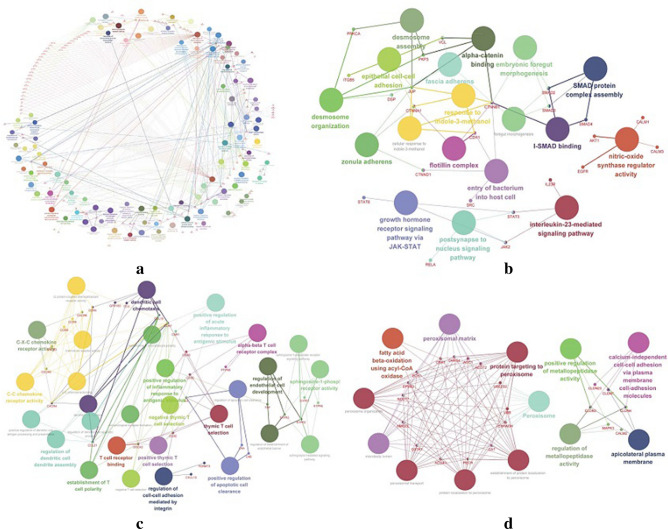
The diagram of GO and KEGG analysis. (**a**) shows the diagram of Turquoise module. (**b**) shows the diagram of Blue module. (**c**) shows the diagram of Brown module. (**d**) shows the diagram of Yellow module.

GO and KEGG enrichment analyses of the obtained modules showed that the turquoise module contained 294 genes, which were mainly related to neutrophil homeostasis, response to mycotoxin, and smooth muscle cell migration (Fig. 3a, Supplementary Table 5). The 67 genes in the blue module were mainly related to transcription coactivator binding and RNA polymerase II repression of TF binding (Fig. 3b, Supplementary Table 6). The 64 genes in the brown module were mainly related to regulation of leukocyte chemotaxis and positive regulation of leukocyte chemotaxis (Fig. 3c, Supplementary Table 7). The 47 genes in the yellow module were mainly related to the apicolateral plasma membrane and regulation of metallopeptidase activity (Fig. 3d, Supplementary Table 8). Therefore, we speculated that the enriched pathways of UC-related modules played roles in the pathogenesis of UC, and part of the pathways were also related to UC classification.

### Identification of hub genes and construction of the PPI and UC multi-factor networks

**Figure 4:**
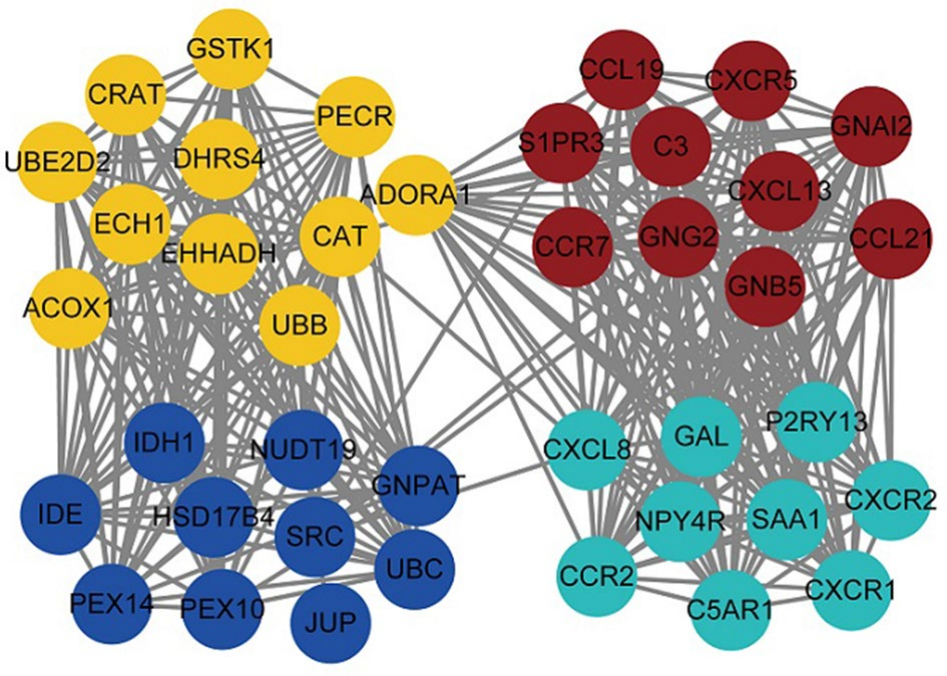
Network diagram of hub gene interactions made by cytoHubba plugin (http://apps.cytoscape.org/apps/cytohubba).

**Figure 5:**
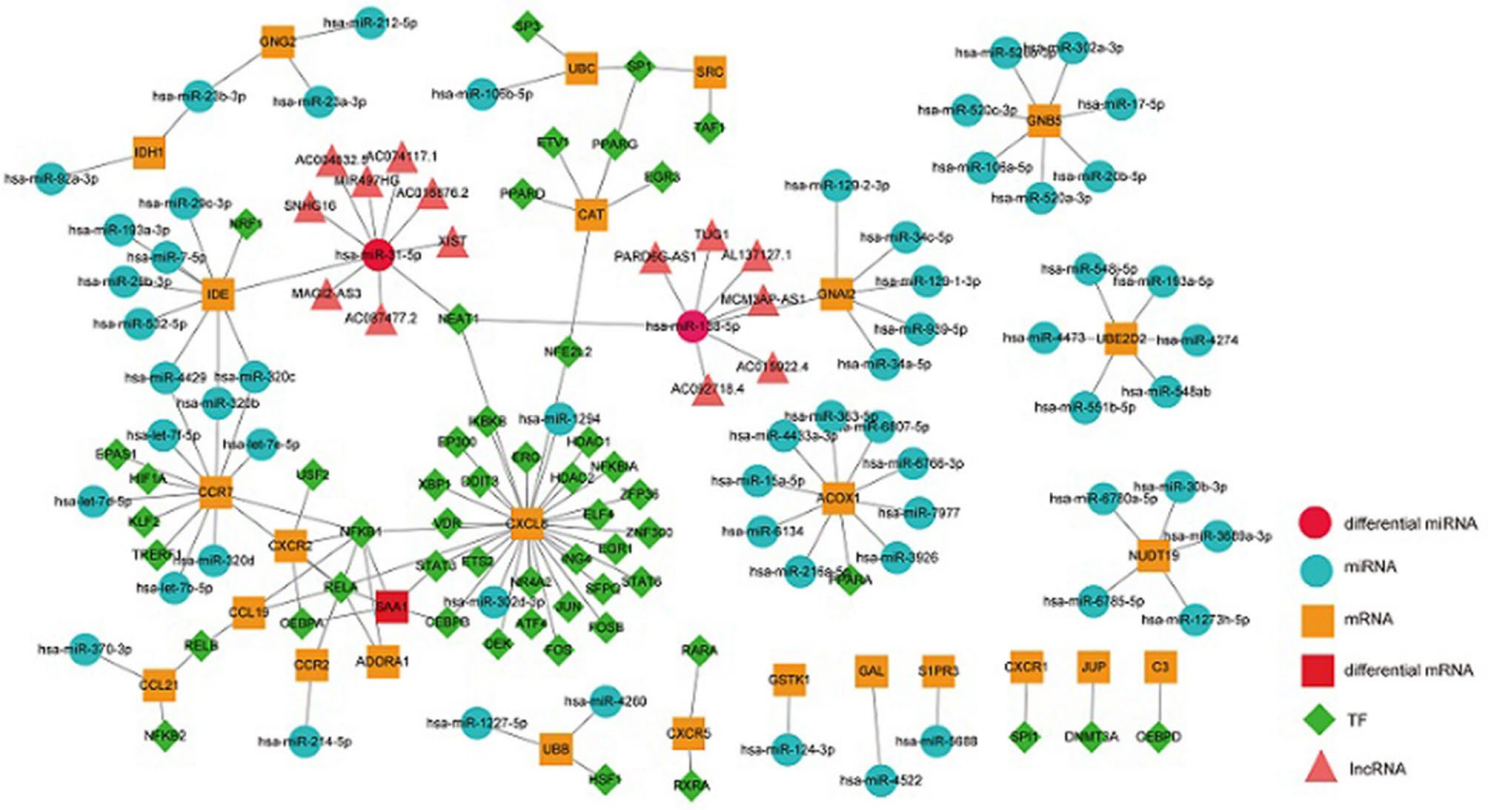
UC multi-factor network diagram made by Cytoscape software 3.8.2 (https://cytoscape.org/).

Ten hub genes from each module were identified, for a total of 40 hub genes, which were then used to construct a PPI network (Fig. 4, Supplementary Table 9). Interactions between miRNA and the hub genes were determined using the miRwalk database, from which 68 pairs of miRNA interactions with the 3′ and 5′ untranslated regions and the coding sequence of the hub genes were identified (Supplementary Table 10). Analysis of the miRNA-expression dataset GSE133059 downloaded from the GEO database indicated two differentially expressed miRNAs overlapping with those interacting with hub genes (hsa-miR-138 and hsa-miR-31-5p) (Supplementary Table 11). These two miRNAs were then used to generate 16 pairs of miRNA–lncRNA interactions (Supplementary Table 12). We then identified 86 regulatory relationships between TFs and hub genes (Supplementary Table 13), followed by merging the identified interactions between miRNA, lncRNA, TF and hub genes to generate the UC multifactor network (Fig. 5).

Additionally, five genes from this network were screened by the Maximal Clique Centrality (MCC) algorithm on Cytohubba, and GO and KEGG analyses revealed their relationship with mitochondrion, responses to hormones, and responses to lipids (Supplementary Table 14). These results showed that metabolic disorders played a key role in the pathogenesis of UC, and some of them might also be related to the classification of UC.

### Identification of module genes for UC disease classification

**Figure 6:**
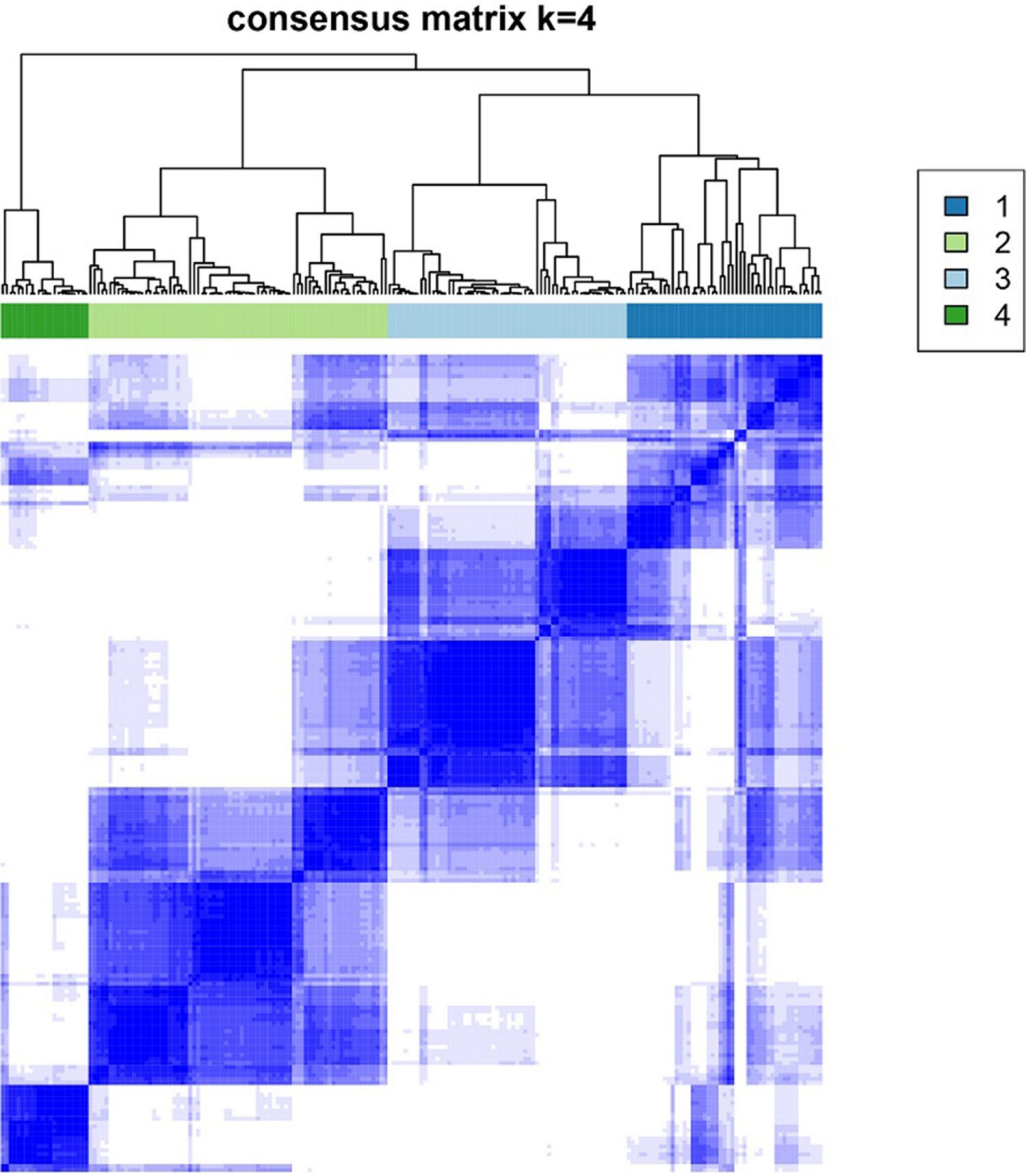
When k = 4, the clustering heat map of UC is made by R package ConsensusClusterPlus (https://bioconductor.org/packages/release/bioc/html/ConsensusClusterPlus.html).

**Figure 7:**
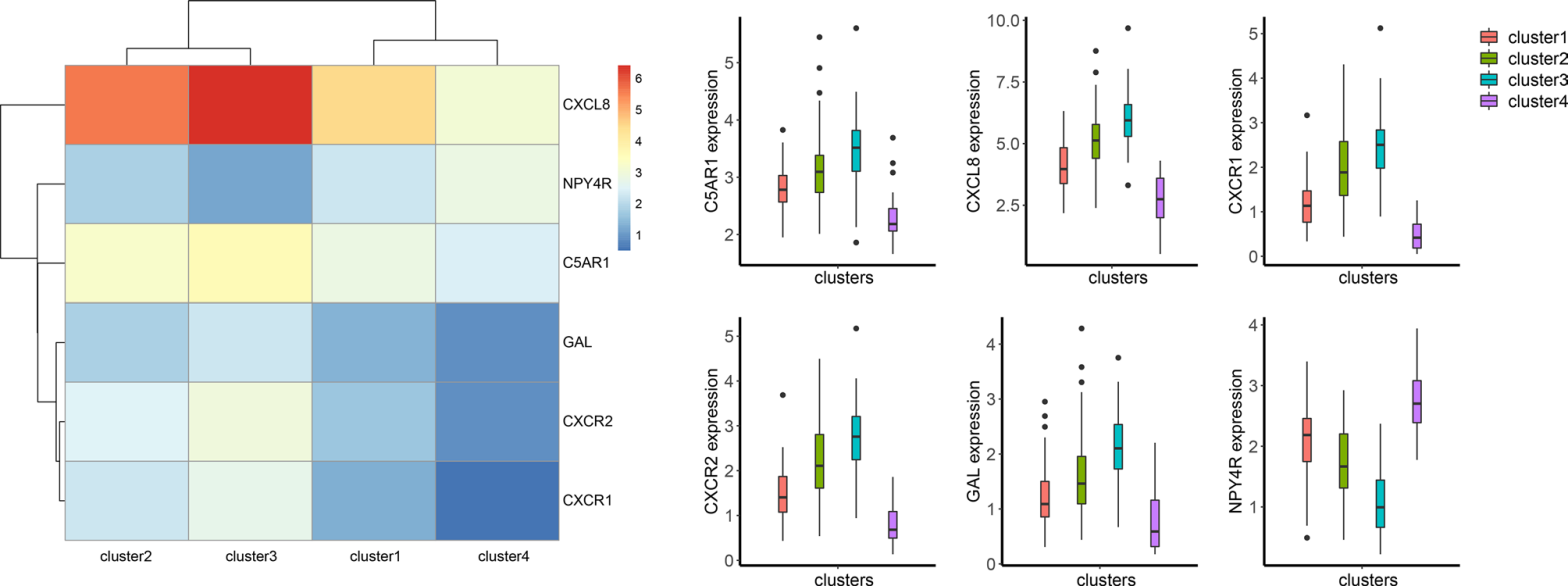
The expression heat map and expression level of 6 hub genes in different clusters, the heat map is made by R package pheatmap (https://cran.r-project.org/web/packages/pheatmap/).

We mapped the 40 hub genes of the modules to the UC patient specimens in GSE109142, followed by clustering of the samples. The results showed that it was appropriate to divide UC disease into four subtypes. Figure 6 shows the hierarchical clustering heatmap of UC across four clusters containing 49, 75, 60, and 22 samples, respectively (Supplementary Table 15). One-way ANOVA identified six genes that showed significant differences in expression between the four groups (*p* <  0.05) (Supplementary Table 16), with Fig. 7 showing a heatmap and box plot of the average expression of these genes. These results indicated that the six identified genes might represent biomarkers for UC subtype classification.

## Discussion

In this study, we identified 60 DEGs between UC patients and healthy individuals based on the GSE109142 and GSE111889 datasets. WGCNA of the DEGs and UC-related genes revealed three functional modules related to UC. The brown and turquoise modules were significantly positively correlated with healthy individuals and negatively correlated with UC patients, whereas the yellow modules were significantly negatively correlated with healthy individuals and negatively correlated with UC patients. Clustering analysis of UC specimens from GSE109142 suggested that UC could be divided into four subtypes, and six hub genes could potentially represent biomarkers for UC classification. However, all potential biomarker genes belong to the turquoise modules. These hub genes included *C-X-C chemokine ligand* (*CXCL*) *8* (also known as *IL-8*), *neuropeptide Y receptor Y4*, *complement C5a receptor 1*, *galanin*, *C-X-C chemokine receptor* (*CXCR*) *2*, and *CXCR1*. Among them, CXCL8 is associated with inflammation grading of intestinal mucosa in UC^22^, and CXCR2 and CXCR1 reportedly play key pathophysiological roles in UC^23^. Additionally, IL-8 reportedly mediates the immune response in UC through CXCR1^24^.

The results of functional enrichment analysis of UC-related modules showed that there was a significant difference in interactions among different modules, which was largely associated with their differing functions. Coincidentally, the six hub genes related to UC classification all belong to the turquoise module. Enrichment analysis of the genes in the turquoise module suggested their involvement in multiple GO pathways, including CXCR activity and binding, with three of the six potential UC classification biomarkers also associated with these pathways. C-X-C chemokines play an important role in leukocyte activation and migration by interacting directly with receptors on the cell surface, including CXCR1 and CXCR2. A previous study described a potential pathological role for C-X-C chemokines in UC^25^, which agrees with the results of GO and KEGG enrichment analyses in the present study and suggests their possibly important role in UC classification. Additionally, the brown module was found to be mainly enriched in pathways associated with leukocyte chemotaxis, and genes in the yellow module were mainly enriched in pathways associated with regulation of metallopeptidase activity. Because leukocyte chemotaxis and metallopeptidase play important roles in chronic inflammation and chronic intestinal tissue destruction in UC^26,27^, we speculated the brown and yellow modules were the most important modules in the pathogenesis of UC.

In addition, by collecting the clinical characteristics of UC patients in 4 subtypes, we obtained the clinical features of different subtypes of UC. UC patients in Cluster 1 had lower Pediatric Ulcerative Colitis Activity Index (PUCAI) scores, lower total Mayo scores, and higher baseline calprotectin levels. UC patients in Cluster 2 had higher PUCAI and total Mayo scores. Most of these patients were treated with cyclosporin as the initial treatment agent. UC patients in this subtype also had higher histology severity scores and lower baseline calprotectin levels, and a week 4 calprotectin levels of ≥ 250. The clinical characteristics of UC patients in Cluster 3 and Cluster 2 were similar, except that UC patients in Cluster3 had higher baseline calprotectin levels. The clinical characteristics of UC patients in Cluster 4 and Cluster1 were similar, except that UC patients in Cluster 4 had lower baseline calprotectin levels.

During construction of the UC multi-factor network, we identified the miRNAs hsa-miR-138-5p and hsa-miR-31-5p, which both interact with hub genes and are differentially expressed between UC and healthy individuals. A previous report showed that hsa-miR-31-5p could serve as an effective biomarker of Crohn’s disease subtypes^28^, and another study identified miR-138-5p as differentially expressed in UC inflamed mucosa relative to non-inflamed mucosa in UC patients and controls^29^. Coincidentally, the UC multi-factor network showed that both miRNAs interact with CXCL5 through the TF nuclear enriched abundant transcript 1 (NEAT1), suggesting that the two miRNAs and NEAT1 could also be used as potential biomarkers for UC classification. However, verification of this hypothesis will require further analysis of UC patient specimens.

This study has some limitations. Because the GSE111889 dataset contains ileum and rectal specimens, and the number of samples from UC patients is small. Therefore, we used both the GSE109142 and GSE111889 datasets for analysis and applied the intersection of the results. To confirm the accuracy of these results, it will be necessary to validate the findings using an appropriate validation dataset. Additionally, our study was limited to in silico prediction. To confirm these results, it will be necessary to verify and extend the findings using a larger cohort of UC patients. We divided UC patients into four groups based on hub genes. However, due to the limited clinical information provided by the samples in the GSE109142 dataset, we cannot relate this classification to the clinical classification of UC well only through the results at this stage. To apply the classification highlighted here as a reference for patient clinical management, it will be necessary to collect and summarize more detailed clinical characteristics of each group. Furthermore, follow-up studies are necessary to clarify whether the other predicted miRNAs, lncRNAs, and TFs activate or inhibit the functional modules chosen for classification, which the in silico analysis in the present study could not determine.

## Conclusion

In this study, we identified 60 DEGs associated with UC two publicly available datasets. A total of 914 UC-related genes were obtained by merging UC-specific DEGs with UC genes from the public database. Additionally, scale-free WGCNA was performed using the 914 UC-related genes, resulting in identification of four modules, three of which were related to UC. A total of 40 hub genes from the modules were selected to construct a multi-factor network, which revealed that UC classification could be accomplished according to four subtypes and six genes, which might represent UC-subtype classification biomarkers.

## Supplementary Information

Supplementary Table 1.

Supplementary Table 2.

Supplementary Table 3.

Supplementary Table 4.

Supplementary Table 5.

Supplementary Table 6.

Supplementary Table 7.

Supplementary Table 8.

Supplementary Table 9.

Supplementary Table 10.

Supplementary Table 11.

Supplementary Table 12.

Supplementary Table 13.

Supplementary Table 14.

Supplementary Table 15.

Supplementary Table 16.

## Data Availability

All the datasets included in this article (GSE109142, GSE111889 and GSE133059) can be downloaded from the GEO database (https://www.ncbi.nlm.nih.gov/geo/).

## Code availability

All the software and websites used in this article have been mentioned in the part of Method.
